# Biliousness

**Published:** 1880-04

**Authors:** 


### BILLIOUSNESS.

Is a greater amount of bile in the blood, than is natural; the result of which is, the eyes and the skin begin to wear a yellow appearance, while various other symptoms manifest themselves according to the temperament, habits and peculiarities of the individual; one has sick headache; another complains of a want of appetite, sometimes loathing the very appearance of food; a third has cold feet and hands; a fourth has chilly sensations, involving the whole body, or running up and down the back; a fifth is costive, women become hysterical and laugh, cry, or talk, while men are moody, pevish, or morose. Bile is naturally of a bright yellow color, but as a man becomes more bilious, it grows darker and is at length as black as tar, causing a state of mind, which the old Romans called atra-bility, "atra" meaning "black"; a scowl is on the countenance, and the person is ilnatured and fretful, finding fault with everybody and everything; hence when a man is cross, he is bilious, and ought to be pitied, and at the same time, be made to take an emetic. The ilnatured are never well, they are "bilious," the system is clogged, the machinery does not work well, and both mind and body are disordered. The safest and best method of getting rid of biliousness is steady work in the open air, for six or eight hours every day, working or exercising to the extent of keeping up a gentle moisture on the skin, this moisture conveys the bile away out of the system, the same result will be accomplished, but not so well, by a good steam bath, or by wrapping up in bed, drinking hot teas, thus "getting up a perspiration," but the atmosphere of the room should be pure, and the diet for several days should consist of coarse bread and fruits. Medicines which "act on the liver" will do the same thing, but they should be advised by the physician, when other means have failed.

The office of the liver is to withdraw the bile from the blood; it is the largest workshop of the body, and is at the right side, about the lower edge of the ribs. When it does not do its work, it is said to be "torpid," asleep, and medicines are given to stimulate it, wake it up, make it act, work faster than common, so as to throw off the excess of bile. When it does not withdraw or separate the bile from the blood, the skin grows yellow, also the whites of the eyes, and the man has the "Yellow Jaundice." When it separates the bile from the blood, but retains it within itself, constipation ensues, appetite is lost, spirits become despondent, and the person is languid, lazy, fretful, and irritable. The liver is in a sense like a sponge, and the bile may be pressed out of it, as water out of a sponge, by pressing the ball of the hand over the region of the liver downwards, from hip to "pit of stomach," two or three minutes at a time, several times a day; this is a good remedy in dyspeptia, and also relieves the stomach of wind, giving immediate and grateful relief sometimes.

